# FEZF1-AS1: a novel vital oncogenic lncRNA in multiple human malignancies

**DOI:** 10.1042/BSR20191202

**Published:** 2019-06-25

**Authors:** Changlong Shi, Li Sun, Yongsheng Song

**Affiliations:** 1 Department of Urology, Shengjing Hospital of China Medical University, Shenyang, Liaoning, China; 2 Department of Breast Surgery, Shengjing Hospital of China Medical University, Shenyang, Liaoning, China

**Keywords:** FEZF1-AS1, LncRNA, Oncogen, Tumor

## Abstract

Long noncoding RNAs (LncRNAs) refer to the RNA with a length of >200 nucleotides, which lack or have no open reading coding frame and have higher tissue and organ specificity compared with the protein coding genes. A surging number of studies have shown that lncRNA is involved in numerous essential regulatory processes, such as X chromosome silencing, genomic imprinting, chromatin modification, transcriptional activation, transcriptional interference and nuclear transport, which are closely related to the occurrence and development of human malignancies. FEZ family Zinc Finger 1-Antisense RNA 1 (FEZF1-AS1) of FEZ family is a recently discovered lncRNA. FEZF1-AS1 is highly expressed in pancreatic cancer, colorectal cancer, lung adenocarcinoma and other human malignancies, and is associated with poor prognosis. As an oncogene, it plays crucial role in the proliferation, migration, invasion and Warburg effect of various tumor cells. In addition, FEZF1-AS1 is also involved in the regulation of multiple signal pathways such as epithelial–mesenchymal transition (EMT), signal transducer and activator of transcription 3 (STAT3) and Wnt/ β-catenin. In this paper, the recent research progress of FEZF1-AS1 in tumorigenesis and development is reviewed systematically.

## Introduction

According to the latest data released by the International Agency for Research on Cancer in September 2018, there were 18.1 million new cancer cases and 9.6 million cancer-related deaths worldwide in 2018, further aggravating the global cancer burden [1]. Although there are currently a variety of treatment options for cancers, the 3- and 5-year survival rates for cancer patients are still poor [2,3]. Most countries still face an absolute increase in the number of cancer patients [1]. Therefore, it is very important to explore and study new biomarkers for clinical diagnosis, treatment and prognosis. Among the more than 3 billion base pairs of human gene sequences, two-third are reversely transcribed, and in the end less than 2% of the nucleic acid sequences are used to encode proteins, most of which do not express proteins, a class of genes known as noncoding RNAs (ncRNAs) [4]. At first, ncRNAs were considered as transcriptional “noise” without specific biological functions [5]. However, with the remarkable progress of genome sequencing technology, an increasing number of ncRNAs have been reported in recent years [6–8]. NcRNAs have been considered to be an indispensable regulatory factor in a series of biological processes, including epigenetics, cell cycle, post-transcriptional regulation, chromatin modification and other aspects [5,9,10]. Long noncoding RNAs (LncRNAs) have been identified as RNA transcripts with more than 200 nucleotides [11]. The number of LncRNAs not only dominates the ncRNAs, but also participates in the regulation of cell differentiation and ontogenesis at multiple levels, and is closely related to many diseases, including tumors [10,12]. FEZ family Zinc Finger 1-Antisense RNA 1 (FEZF1-AS1), as a newly discovered lncRNA, has a lot of evidence that it acts as oncogene. It is significantly expressed in pancreatic cancer (PC), ovarian cancer, nasopharyngeal carcinoma (NPC), hepatocellular carcinoma (HCC), cervical cancer (CC), colorectal cancer (CRC), multiple myeloma (MM), breast cancer ( BC), osteosarcoma (OS), non-small-cell lung cancer (NSCLC), gastric cancer (GC) and other malignancies, and is closely related to the occurrence and progress of a variety of malignancies. In this paper, the recent research progress on the biological function, mechanism and clinical significance of FEZF1-AS1 in malignancies is reviewed systematically.

## Characteristics and discovery of FEZF1-AS1

**Figure 1:**
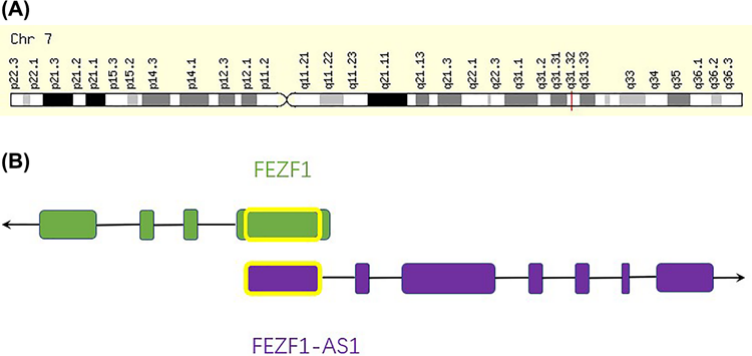
Schematic diagram of the location of FEZF1-AS1 and its relationship with FEZF1 (**A**) FEZF1-AS1 location and (**B**) Schematic diagram of the sequence relationship between FEZF1-AS1 and FEZF1 gene (arrow for transcription direction, yellow border for overlapping region).

FEZF1-AS1 is located on chromosome 7q31.32 (Figure 1), with a length of 2653bp. In 2004, Japanese researchers first discovered full-length CDNA in isolated human beings [13]. Genomic structure analysis demonstrated that it contained three splicing variants (FEZF1-AS1-201, FEZF1-AS1-202, FEZF1-AS1-203) and seven “exons”. The first exon and the first exon of FEZF1mRNA, had 611 complete complementary nucleotides, so it was called FEZF1-AS1 (Figure 1). FEZF1 is a transcriptional inhibitor of zinc finger double domain protein family. It contains 4 exons, 2105 bases, 475 amino acids and 6 C2-H2 zinc finger domains. Many years of research have shown that FEZF1 plays a key role in the development of nervous system [14]. Recently, FEZF1 has also been implicated in the progression of tumorigenesis, including enhancing the proliferation and tumorigenicity of GC by binding and activating the oncogene K-ras [15], promoting cell migration and invasion of CRC cells [16], playing a carcinogenic role in CC by acting as a transcriptional activating factor in the Wnt pathway [17], facilitating the development of glioma cells by activating Akt-ERK pathway [18], and so on. Based on the enthusiasm for the study of the FE1 family, there are more and more reports about FEZF1-AS1. Chen et al. [16] first reported that FEZF1-AS1 was up-regulated in human primary CRC and was associated with CRC metastasis and poor prognosis. Moreover, the expression of FEZF1-AS1 affected the proliferation, migration and invasion of CRC cells. In addition, there was a positive correlation between FEZF1-AS1 and FEZF1 expression in CRC. Subsequently, a growing number of studies have found that FEZF1-AS1 is up-regulated in PC, ovarian cancer, NPC, HCC, CRC, MM, BC, OS, NSCLC, GC and other malignancies. The expression of FEZF1-AS1 is closely related to the TNM stages and overall survival of HCC, PC, CRC, BC, OS, NSCLC and GC, as well as the poor prognosis of various malignancies, including PC, CC, lung adenocarcinoma (LAD) and GC. Again, knockdown of FEZF1-AS1 inhibited the proliferation, migration and invasion of HCC, CRC, OS, BC, LAD, PC and NSCLC. FEZF1-AS1 also promoted Warburg effects in PC and CRC cells. This review summarizes the latest evidence related to the abnormal expression of FEZF1-AS1 in human malignancies, its potential molecular mechanism and clinical significance (Tables 1 and 2), and are described in detail below.

**Table 1:** Functional characterizations of FEZF1-AS1 in multiple human cancers

Cancer types	Expression	Role	Biological function	Related genes	References
Pancreatic cancer	Up-regulated	Oncogenic	Cell proliferation, apoptosis, migration, invasive, progression, Warburg effect, cell cycle control	HIF-1α, VEGF, *miR-142*, EGFR, AKT, p-AKT, *miR-107, miR-133a*, FEZF1	[20,21]
Ovarian cancer	Up-regulated	Oncogenic	Cell proliferation, apoptosis	STAT3, p-STAT3	[23]
Nasopharyngeal carcinoma	Up-regulated	Oncogenic	Cell proliferation, migration, invasive, cell cycle control, EMT	P21, CyclinD1, E-cadherin, Vimentin, N-cadherin, Wnt/β-catenin	[25]
Hepatocellular carcinoma	Up-regulated	Oncogenic	Cell proliferation, migration, invasive, cell cycle control, EMT	JAK/STAT3, E-cadherin, N-cadherin, Vimentin	[28]
Cervical cancer	Up-regulated	Oncogenic	No description	No description	[32,33]
Colorectal cancer	Up-regulated	Oncogenic	Cell proliferation, apoptosis, migration, invasive, progression, Warburg effect, cell cycle control	PKM2, STAT3, FEZF1MCL1, BIRC5, CCND1, BCL2L1, CDH1, MMP2, MMP9	[16,35]
Multiple myeloma	Up-regulated	Oncogenic	Cell proliferation, cell cycle control, apoptosis	*miR-610*, AKT3	[37]
Breast cancer	Up-regulated	Oncogenic	Cell proliferation, migration, invasive, progression	*miR-30a*, Nanog, Oct4, SOX2	[39]
Osteosarcoma	Up-regulated	Oncogenic	Cell proliferation, migration, invasive, progression	*miR-4443*, NUPR1	[41]
Non-small-cell lung cancer	Up-regulated	Oncogenic	Cell proliferation, migration, invasive, progression, EMT cell cycle control	CDK2, CDK4, CDK6, cleaved-caspase3, total-caspase3, cleaved-caspase9, total-caspase9, cleaved-PARP, P57, LSD1, EZH2, Slug, total-PARP, Vimentin, HUR, FEZF1, ZO-1, Snail, Twist, AXIN1, E-cadherin, β-catenin TCF4	[44–47]
Gastric cancer	Up-regulated	Oncogenic	Cell proliferation, apoptosis, progression, cell cycle control	Wnt/β-catenin, p21, CDK2, CDK4, CDK6, LSD1, CyclinD1, SP1, E-cadherin, H3K4me3	[49–51]

**Table 2:** Clinical features of FEZF1-AS1 in multiple human cancers

Cancer types	Clinicopathological features	References
Pancreatic cancer	Poorer overall survival, positive lymph node metastasis, advanced TNM and AJCC stages, positive neural invasion	[20,21]
Ovarian cancer	Poorer overall survival	[23]
Nasopharyngeal carcinoma	Poorer overall survival and relapse-free survival, positive distant metastasis	[25]
Hepatocellular carcinoma	Poorer overall survival, advanced TNM stages, larger tumor size, positive venous invasion	[28]
Cervical cancer	Poorer overall survival, advanced FIGO stages, poorer histological grade, positive distant metastasis	[32,33]
Colorectal cancer	Poorer overall survival and relapse-free survival, higher T-stage, positive lymph node metastasis and distant metastasis	[16,35]
Multiple myeloma	No description	
Breast cancer	Poorer overall survival	[39]
Osteosarcoma	Poorer overall survival, later Clinical stage	[41]
Non-small-cell lung cancer	Poorer overall survival, advanced TNM stages, poorer differentiation, larger tumor size, positive lymph node metastasis, tumor family history, poorer histological grade	[44–47]
Gastric cancer	Poorer overall survival and relapse-free survival, larger tumor size, advanced TNM and AJCC stages, higher grade	[49–51]

## FEZF1-AS1 in various human malignancies

### PC

PC is a malignancy with high parallel morbidity and mortality and poor prognosis [19]. Chen et al. found a large number of differentially expressed lncRNAs in pancreatic ductal adenocarcinoma tissues and non-tumor tissues through genechip analysis in 2014, among which the expression of FEZF1-AS1 was the most obvious. Ye et al. [20] then confirmed that FEZF1-AS1 and FEZF1 were markedly expressed in pancreatic ductal adenocarcinoma tissues and cell lines, compared with paired adjacent normal PC tissues and human pancreatic ductal epithelial cell lines (HPDE6-C7) by quantitative real-time PCR (qRT-PCR) and fluorescence *in situ* hybridization (FISH). FEZF1-AS1 expression was increased in patients with poor differentiation, advanced AJCC stages and positive nerve invasion [20]. Survival analysis showed that up-regulation of FEZF1-AS1 was significantly correlated with overall survival, and univariate and multivariate Cox regression analysis demonstrated that overexpression of FEZF1-AS1 and FEZF1 was markedly correlated with increased mortality of tumor patients, and was an essential factor affecting prognosis [20]. The expression of FEZF1 in PC tissues was positively correlated with the transcription level of FEZF1-AS1. Functional experiments confirmed that FEZF1-AS1 could bind to *miR-107* as a competing endogenous RNA (ceRNA), thereby regulating the expression of FEZF1. The FEZF1-AS1/miR-107/FEZF1 axis plays a key role in the proliferation, apoptosis, migration and invasion of pancreatic ductal adenocarcinoma cells [20]. In addition, knockdown of FEZF1-AS1 or FEZF1 leads to a significant reduction in extracellular oxidative phosphorylation (ECAR) and inhibits glycolysis in pancreatic ductal adenocarcinoma cells [20]. This suggests that the FEZF1-AS1/*miR-107*/FEZF1 axis promotes the Warburg effect in pancreatic ductal adenocarcinoma cells.

Qu et al. [21] also demonstrated that FEZF1-AS1 is more markedly expressed in pancreatic ductal adenocarcinoma tissues and cell lines. FEZF1-AS1 is more commonly expressed in patients with advanced TNM stage (II), larger tumors size and positive lymph node metastasis [21]. Kaplan–Meier analysis showed that patients with low FEZF1-AS1 expression had a longer overall survival. Univariate Cox regression analysis displayed that FEZF1-AS1 expression and lymph node metastasis might be risk factors affecting overall survival, while multivariate Cox regression analysis further revealed that high expression of FEZF1-AS1 was a risk factor affecting overall survival [21]. Further studies illustrated that FEZF1-AS1 could bind to *miR-142* and *miR-133a*, and regulate the proliferation and invasion of PC cells through the *miR-142*/HIF1α axis under hypoxia and the *miR-133a*/epidermal growth factor receptor (EGFR) axis under normoxic conditions [21].

### Ovarian cancer

As the eighth most common cancer, ovarian cancer has become the most lethal gynecological malignant tumor in women [22]. Zhao et al. [23] confirmed the high expression of FEZF1-AS1 in ovarian cancer tissues and cell lines by qRT-PCR, compared with adjacent non-tumor tissues and normal ovarian cell line (FTE187). Survival analysis demonstrated that up-regulation of FEZF1-AS1 in ovarian cancer patients was associated with poor prognosis [23]. After knockdown of FEZF1-AS1, more ovarian cancer cells entered the G0/G1 phase, which inhibited the proliferation of ovarian cancer cells and increased the proportion of ovarian cancer cells apoptosis [23]. Further studies have identified that FEZF1-AS1 activated the Janus Kinase (JAK)-signal transducer and activator of transcription 3 (STAT3) signaling pathway by regulating the phosphorylation of STAT3, promoting the proliferation of ovarian cancer cells and inhibiting apoptosis [23].

### NPC

NPC is a rare malignancy in most regions of the world, but it is very common in China [24]. Cheng et al. [25] reported that FEZF1-AS1 was elevated expressed in NPC tissues and cell lines compared with pericarcinomatous tissue and human nasopharyngeal epithelial cell line (NP69). FEZF1-AS1 expression is closely related to the overall survival rate, disease-free survival rate and distant metastasis of NPC patients [25]. Functional experiments showed that the knockdown of FEZF1-AS1 could significantly induce the G0/G1 block of NPC cells, reduce the migration and invasion ability of NPC cells, and signally inhibit the growth of tumor in nude mice [25]. Again, after the silencing of FEZF1-AS1, the expressions of E-cadherin and P21 in NPC cells were memorably increased, while the expressions of N-cadherin, Vimentin, β-catenin and CyclinD1 were obviously decreased [25]. This suggests that FEZF1-AS1 has an effect on EMT and WNT/β-catenin pathways in NPC cells. Several studies have shown that the WNT/β-catenin signaling pathway is an important way to activate EMT [26], and FEZF1-AS1 encourages β-catenin entering the nucleus, which may be an important step in the induction of EMT in nasopharyngeal carcinoma.

### HCC

HCC is one of the most common and fatal cancers in the world [27]. Wang et al. [28] detected the expression of FEZF1-AS1 in human HCC tissues and cell lines by qRT-PCR, which was significantly amplified than that in paired paracancerous tissues and cell lines. The expression of FEZF1-AS1 was closely related to the size of HCC, TNM stages of tumor and venous invasion [28]. Kaplan–Meier analysis revealed that high expression of FEZF1-AS1 was associated with pooper overall survival in patients with hepatoma [28]. Function experiments illustrated that after FEZF1-AS1 knockdown, the number of HCC cells in G0/G1 phase increased, the number of S phase cells decreased, cell proliferation rate decreased, migration, invasion and growth ability in nude mice decreased [28]. JAK2/STAT3 pathway plays an indispensable role in EMT of HCC cells [29,30]. Western blot experiments confirmed that knockdown FEZF1-AS1 inhibited the expression of N-cadherin and Vimentin, facilitated the expression of E-cadherin protein and inhibited the EMT process [28]. Further studies revealed that FEZF1-AS1 promotes cell invasion and EMT through the JAK2/STAT3 signaling pathway in human HCC [28].

### CC

CC is the third most common malignant tumor among women worldwide [31]. Chen et al. [32] reported the obvious elevated expression of FEZF1-AS1 in CC for the first time using lncRNA genechip analysis and qRT-PCR. Zhang et al. [33] used qRT-PCR to find that the expression of FEZF1-AS1 in CC tissue was higher than that in normal cervical tissue adjacent to cancer. The expression of FEZF1-AS1 in CC tissues was related to high level histological grading, distant metastasis and FIGO stages [33]. Kaplan–Meier analysis found that the overall survival of patients with high FEZF1-AS1 expression was lower than that of patients with low expression [33]. In univariate Cox regression analysis, FEZF1-AS1 was associated with poor survival, and multivariate Cox regression analysis confirmed that FEZF1-AS1 expression was an independent prognostic factor affecting overall survival [33].

### CRC

CRC is one of the most common malignancies in the world [34]. Chen et al. [16] applied qRT-PCR to detect that the expression of FEZF1-AS1 in CRC tissues of group 34 was higher than that in adjacent normal tissues, and the same was accounted in CRC cell lines. Up-regulation of FEZF1-AS1 is closely related to advanced T-stage, positive lymph node metastasis and distant metastasis of CRC [16]. Kaplan–Meier analysis detected that the overexpression of FEZF1-AS1 in CRC was correlated with the pooper overall survival and relapse-free survival, univariate and multivariate Cox regression analysis showed that the high expression of FEZF1-AS1 was an independent prognostic factor of CRC [16]. Function experiments demonstrated that the silencing of FEZF1-AS1 mainly led to the increase of G0/G1 phase and the decrease of S phase in CRC cells, and the tumor tissue proliferation ability of FEZF1-AS1 knockdown was lower than that of the control cells, the migration and invasion ability of CRC cells with FEZF1-AS1 knockdown was reduced [16]. In addition, silencing of FEZF1-AS1 inhibited tumor growth and metastasis in nude mice. Further experiments displayed that inhibition of FEZF1-AS1 expression decreased the expression of homologous gene FEZF1mRNA and protein, and the expression of FEZF1-AS1 and FEZF1 was positively correlated [16].

Bian et al. [35] finally selected 52 lncRNAs with low expression in normal colorectal tissues and high expression in CRC tissues through genechip analysis, especially FEZF1-AS1. Furthermore, qRT-PCR confirmed that the expression of FEZF1-AS1 in CRC tissues was significantly amplified than that in adjacent tissues. The expression of FEZF1-AS1 in CRC was positively correlated with tumor stages [35]. Survival analysis revealed that up-regulation of FEZF1-AS1 was associated with poorer overall survival and relapse-free survival. Again, the expression of FEZF1-AS1 was an independent prognostic factor for CRC, and the overexpression of FEZF1-AS1 brought worse prognosis to patients [35]. FEZF1-AS1 with relatively high expression in CRC cells was screened for experiments, and it was confirmed that silencing of FEZF1-AS1 could reduce the S phase of CRC cells and increase apoptosis, transwell analysis illustrated that FEZF1-AS1 knockdown inhibited the migration and invasion of CRC cells, and tumor xenograft model in nude mice demonstrated that FEZF1-AS1 overexpression stimulated lung metastasis and liver metastasis of CRC [35]. RNA pull-down assay and mass spectrometry analysis predicted that pyruvate Kinase M 2 (PKM2) was a related protein of FEZF1-AS1, biological information analysis found that STAT3 could be regulated by FEZF1-AS1 and PKM2 [35]. Further studies have identified that FEZF1-AS1 enhances the stability of CRC cells by binding to PKM2 and increases the activity of PKM2, which leads to the enhancement of aerobic glycolysis of CRC cells and activates the STAT3 signaling pathway to facilitate the proliferation and metastasis of CRC cells [35].

### MM

MM, an incurable plasma cell malignancy, is the second most common hematologic cancer in the United States [36]. Li et al. [37] confirmed through qRT-PCR that the expression of FEZF1-AS1 in MM tissues and cell lines was markedly overexpressed than that in adjacent normal tissues and human normal plasma cells (nPCs). Function experiments showed that knockdown of FEZF1-AS1 prevented MM cells from transforming from G1 phase to S phase, reduced proliferation of MM cells and increased apoptosis [37]. Further studies revealed that *miR-610* was the target of FEZF1-AS1 in MM, and the expression of AKT3mRNA was increased and negatively correlated with the expression of *miR-610* [37]. Silencing FEZF1-AS1 could decrease the expression of AKT3mRNA and protein, while FEZF1-AS1 was positively correlated with the expression of AKT3 [37]. This suggests that FEZF1-AS1 binds to *miR-610* as ceRNA and regulates the *miR-610*/AKT3 axis to promote the proliferation of MM cells.

### BC

BC is the most common malignancy among women worldwide and is one of the main causes of cancer patients’ death [38]. Zhang et al. [39] exerted qRT-PCR detection to find that FEZF1-AS1 was distinctly expressed in BC tissues and cell lines compared with pericarcinomatous tissues and human normal breast epithelial cells (MCF-10a). Survival analysis showed that the overall survival of BC patients with high FEZF1-AS1 level was lower than that of BC patients with low FEZF1-AS1 level [39]. Flow cytometry and sphere formation assays indicated that FEZF1-AS1 can reduce the CD44^+^/CD24^-^ rate and mammosphere-forming ability in Breast cancer stem-like cells (BCSC), and a series of functional experiments indicated that, compared with the control group, knockdown of FEZF1-AS1 can reduce expression of stem factors (Nanog, Oct4, Sox2) and inhibit proliferation, migration, invasion, and growth in vivo of BCSC. Further studies demonstrated that FEZF1-AS1 regulated the expression of Nanog protein by binding *miR-30a*, and formed the pathway of FEZF1-AS1/*miR-30a*/Nanog to accelerate the the progress of BC [39].

### OS

OS is one of the most aggressive and common malignant bone tumors, often occurring in adolescents [40]. Zhou et al. [41] used qRT-PCR to detect and found that FEZF1-AS1 was obviously expressed in OS tissues and cell lines. Statistical analysis showed that FEZF1-AS1 was excessive expressed in the metastatic group compared with the non-metastatic group [41]. In addition, the expression of FEZF1-AS1 in stage III OS was higher than that in stage I/II [41]. Kaplan–Meier analysis displayed that lower FEZF1-AS1 meant poorer patient survival and an advanced phenotype of OS [41]. Function experiments revealed that after silencing of FEZF1-AS1, the proliferation, migration and invasion abilities of OS cells were inhibited, while the up-regulation was on the contrary [41]. Tumor xenograft model in nude mice illustrated that the absence of FEZF1-AS1 delayed the growth of OS *in vivo*, reduced the size of tumor and inhibited the number of lung metastases [41]. Further studies demonstrated that *miR-4443* was a potential binding miRNA of FEZF1-AS1, while there was a potential binding site of *miR-4443* in the 3-utr of NUPR1mRNA. FEZF1-AS1 stimulates the progression of OS by binding *miR-4443* to regulate the *miR-4443*/NUPR1 axis [41].

### NSCLC

Lung cancer is one of the most common causes of cancer deaths worldwide [42]. NSCLC is the main subtype of lung cancer, accounting for about 80–85% of new cases of lung cancer [43]. He et al. [44] confirmed through qRT-PCR that FEZF1-AS1 was signally expressed in NSCLC tissues and cell lines compared with normal para-tumor tissues and human normal lung epithelial cells (16HBE). Statistical analysis showed that the high expression of FEZF1-AS1 was closely related to positive lymph node metastasis, low differentiation grade and advanced TNM stages [44]. Function experiments displayed that the proliferation, migration and invasion abilities of NSCLC cells were reduced after the knockdown of FEZF1-AS1 [44]. Further studies revealed that FEZF1-AS1 enhanced EMT by inhibiting E-cadherin and regulating the Wnt/β-catenin pathway in NSCLC [44]. Again, RNA immunoprecipitation (RIP) and Chromatin immunoprecipitation (ChIP) experiments found that FEZF1-AS1 can directly bind Enhancer of Zeste Homolog 2 (EZH2) and Lysine-Specific Demethylase 1 (LSD1), while EZH2 and LSD1 can combine with promoter regions of E-cadherin to exert demethylation effect, that is, silencing FEZF1-AS1 can up-regulate the expression of E-cadherin [44]. The down-regulation of FEZF1-AS1 expression can increase the expression of AXIN1 and reduce the expression of β-catenin, indicating that FEZF1-AS1 can regulate the WNT/β-catenin signal pathway in NSCLC and participate in the growth of NSCLC [44].

Gong et al. [45] applied qRT-PCR to detect 160 cases of NSCLC and its adjacent tissues, and found that compared with adjacent tissues, FEZF1-AS1 was significantly highly expressed in tumor tissues, and related to advanced TNM stages and tumor family history. In addition, they found that FEZF1-AS1 was highly correlated with FEZF1 in NSCLC [45].

Jin et al. [46] reported that FEZF1-AS1 was dramatically expressed in LAD tissues and cell lines, compared with normal tissues adjacent to tumor and human normal lung epithelial cells (BEAS-2B). Statistical analysis illustrated that high level of FEZF1-AS1 was correlated with larger tumor size, advanced TNM stages of tumor and positive lymph node metastasis [46]. Kaplan–Meier analysis demonstrated that overexpression of FEZF1-AS1 was associated with poor prognosis of patients. Cox regression model analysis showed that high levels of FEZF1-AS1 could be used as a prognostic factor [46]. Function experiments displayed that FEZF1-AS1 knockdown could block LAD cells cycle in G1 phase, inhibit their proliferation and facilitate their apoptosis [46]. Further studies revealed that FEZF1-AS1 could simultaneously recruit EZH2 and LSD1 promoter regions to P57, and inhibit their transcription, thus promoting the occurrence and development of LAD [46].

Liu et al. [47] also reported that FEZF1-AS1 was observably expressed in LAD tissues and cell lines. Statistical analysis illustrated that the expression of FEZF1-AS1 in LAD tissues was related to histological grading and lymph node metastasis [47]. Kaplan–Meier analysis demonstrated that the expression of FEZF1-AS1 was related to the overall survival of patients, and the elevated expression of FEZF1-AS1 predicted poor prognosis of patients [47]. Function experiments revealed that knockdown of FEZF1-AS1 inhibited the proliferation, migration and invasion of LAD cells [47]. Further study accounted that FEZF1-AS1 was positively correlated with the expression of FEZF1, and FEZF1-AS1 it was shown in LAD cells that FEZF1-AS1 played the role of oncogene at least partly by regulating FEZF1 [47].

### GC

GC is the fourth most common cancer in the world and the second leading cause of death among cancer patients [48]. Gu et al. [49] obtained the expression profiles of IncRNA and mRNA in GC by high-throughput sequencing in three patients with gastric adenocarcinoma. The expression difference of IncRNAs and mRNAs in GC tissues and adjacent normal tissues was determined, and the co-expression network of IncRNA and mRNA was constructed [49]. At the same time, using The Cancer Genome Atlas (TCGA) database analysis and other methods to verify, and finally identified nine potential diagnostic value for gastric adenocarcinoma IncRNA, including FEZF1-AS1 [49].

Liu et al. [50] conducted genechip analysis and identified that the expression of FEZF1-AS1 in GC tissues was higher than that in non-cancerous tissues. By qRT-PCR, it was confirmed that FEZF1-AS1 was observably expressed in GC tissues and cell lines [50]. The up-regulation of FEZF1-AS1 in GC is closely related to larger tumor size and advanced TNM stages. Survival analysis displayed that the prognosis of patients with high FEZF1-AS1 expression was lower than that of patients with low FEZF1-AS1 expression [50]. Function experiments illustrated that GC cells were blocked in G1-S phase after silencing of FEZF1-AS1, and the cell cycle was reduced in S phase, the proliferation ability was reduced, and the proportion of early and late apoptosis was increased [50]. GC cells transfected with sh-FEZF1-AS1 were injected into 14 nude mice subcutaneously. Two weeks later, it was found that tumors in the sh-FEZF1-AS1 group were smaller in size and lighter in mass [50]. It is suggested that FEZF1-AS1 plays a vital role in tumorigenesis and tumor growth of GC. Further studies demonstrated that LSD1 can directly bind to the promoter region of P21 and mediate modification of Histone H3lysine-4 di-methylation (H3K4me2), while knockdown of FEZF1-AS1 reduced LSD1 and enhanced demethylation ability of H3K4me2, but had no obvious effect on H3K4me1 [50]. These data indicate that FEZF1-AS1 inhibits p21 transcription by recruiting LSD1, leading to H3K4me2 demethylation of p21 promoter in GC to encourage the proliferation of GC. In addition, the study confirmed that the expressions of FEZF1-AS1 and SP1 were positively correlated in GC tissues. SP1 can directly bind to the promoter region of FEZF1-AS1 and induce FEZF1-AS1 transcription to promote the proliferation of GC cells [50].

Wu et al. [51] confirmed through qRT-PCR that FEZF1-AS1 was evidently expressed in GC tissues and cell lines. High expression of FEZF1-AS1 was associated with high tumor grade and advanced stages [51]. Kaplan–Meier analysis showed that overexpression of FEZF1-AS1 was related to pooper overall survival and relapse-free survival. The results of Receiver Operating Characteristic (ROC) curve analysis displayed that FEZF1-AS1 had high sensitivity and specificity in the differential diagnosis of GC and adjacent non-tumor tissues [51]. Further experiments revealed that the viability of GC cells decreased after FEZF1-AS1 silencing, the results of flow cytometry illustrated that GC cells stagnated in G0/G1 phase. The number of apoptotic cells increased. The results of Western blot demonstrated that the expressions of β-catenin, c-myc and cyclinD1 were reduced, and the expressions of E-cadherin were increased [51]. This suggests that FEZF1-AS1 may promote the occurrence and development of GC by activating the Wnt/ β-catenin signal pathway.

## Underlying molecular mechanisms of lncRNA and FEZF1-AS1

**Figure 2:**
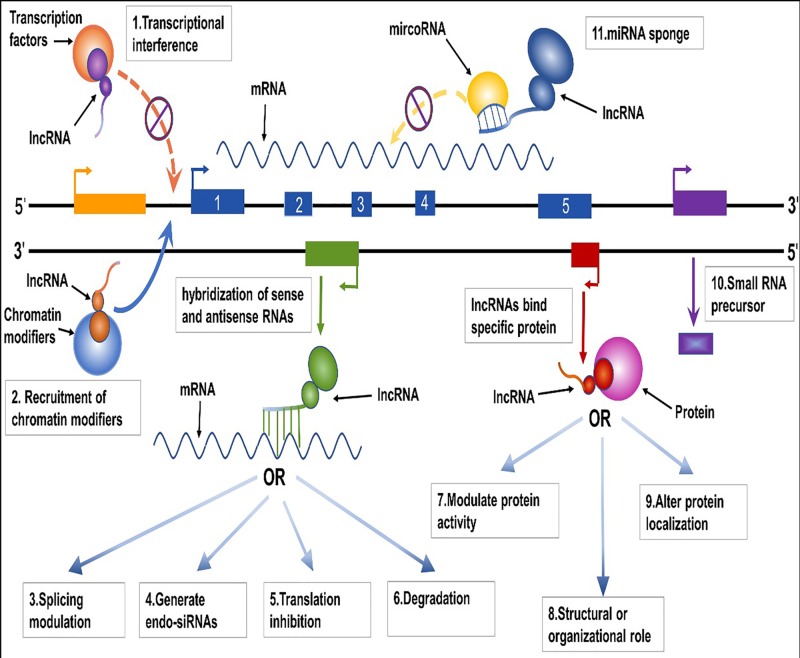
Potential Molecular Mechanism of lncRNA The molecular mechanism of lncRNA is as follows: (**1**) Binding transcription factors interfere with their binding to the upstream promoter region (orange) and regulate the expression of downstream genes (blue). (**2**) Recruitment of chromatin modifiers to change the level of chromosome modification, thus affecting the transcription and expression of genes. (**3**) A complementary double strand is formed with the transcript of the protein gene, which affects its splicing. (**4**) It forms complementary double strands with the transcripts of protein-encoding genes and produces endogenous siRNA under the action of Dicer enzyme. (**5**) It forms a complementary double strand with the transcript of the protein gene and suppresses its translation. (**6**) It forms complementary double strands with the transcripts of protein genes, which affect its stability. (**7**) Bind to specific proteins to regulate their activity. (**8**) As a scaffold or bridge for protein interaction, it affects the formation of protein polymers. (**9**) Binding to specific proteins to change their cellular localization. (**10**) As the precursor of small molecule RNA. (**11**) The adsorption of miRNA, inhibited its binding to mRNA, which prevented the degradation of mRNA.

**Figure 3:**
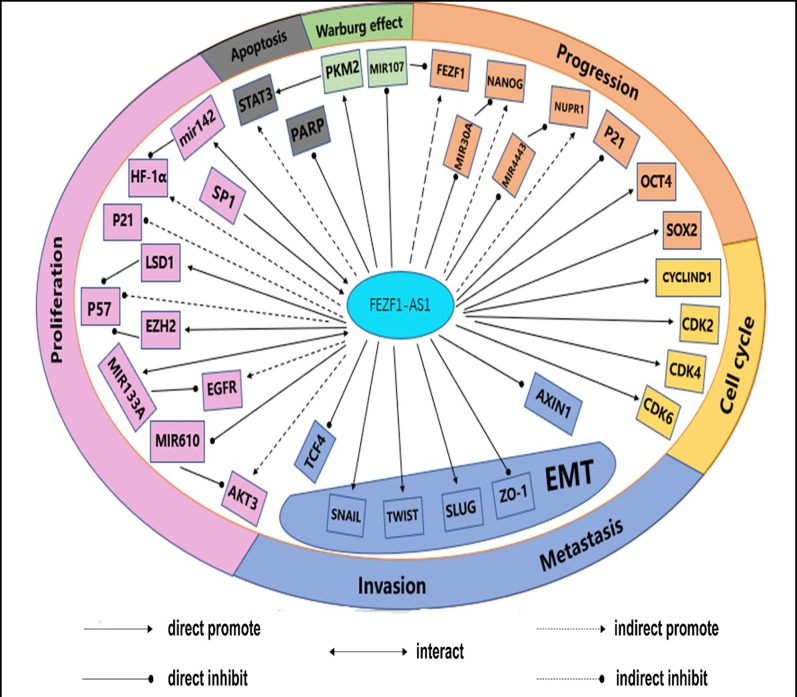
The relationship between the effects of FEZF1-AS1 on tumor cell cycle control, proliferation, apoptosis, metastasis, invasion and Warburg effect

In the early stage, it was considered that *in situ* regulation was the only mechanism of lncRNA. With the deepening of research, it was found that the remote regulation mechanism of lncRNA existed widely in organisms. The mechanism of action is summarized in Figure 2. FEZF1-AS1 perfectly demonstrates part of the mechanism of lncRNA. As shown in Figure 3, FEZF1-AS1 is involved in the proliferation, apoptosis, migration, invasion and Warburg effect of a variety of malignant tumors, thus playing a role in the occurrence and development of malignancies.

### Transcriptional regulation

LSD1 can demethylate the single and dimethylated residues of lysin-4 on histone H3 [52]. Various experiments have proved that FEZF1-AS1 is involved in the regulation of P21, H3K4me2 and LSD1. FEZF1-AS1 can inhibit the transcription of P21 by modifying the H3K4me2 of P21 promoter region to accelerate the progression of tumor. In addition, Liu et al. [50] also reported that SP1 can directly bind to the promoter region of FEZF1-AS1 and induce FEZF1-AS1 transcription in GC cells. P57 is a direct target of EZH2 and is inhibited by a variety of epigenetic mechanisms in ovarian cancer, BC and NSCLC [53–55]. Jin et al. [46] proved that FEZF1-AS1 regulates P57 in LAD cells mainly by recruiting RNA binding proteins EZH2 and LSD1 into the promoter region of P57 and inducing histone modification. He et al. [44] found that FEZF1-AS1 knockdown in NSCLC reduces the binding ability of EZH2 and LSD1 to the promoter region of E-cadherin, inhibits the transcription of E-cadherin and affects EMT.

### EMT pathway

EMT plays a central role in various pathological processes, including wound healing, tissue fibrosis and tumor progression [56]. Matrix Metallopeptidase 2 (MMP2), Matrix Metallopeptidase 9 (MMP9), E-cadherin, N-cadherin, Vimentin, Integrin β-1, Twist, Zonula Occluden-1 (ZO-1), Slug and Snail are indispensable markers in the EMT process [56]. A number of studies have shown that the expression of FEZF1-AS1 is positively correlated with the expression of Slug, Snail, Twist, Vimentin, N-cadherin, MMP2 and MMP9, while negatively correlated with the expression of E-cadherin and ZO-1. Studies have demonstrated that different combinations of various pathways play a key role in the induction of EMT [57]. FEZF1-AS1 can activate EMT of tumor cells through Wnt/ β-catenin and JAK2/STAT3 signaling pathways.

### Wnt/β-catenin pathway

Wnt signaling pathway plays an important role in the occurrence, development and metastasis of different types of tumors [58]. β-catenin is a component of the cadherin complex, that controls cell–cell adhesion [59]. Cheng et al. [25] reported that the Wnt/ β-catenin signal pathway also plays a crucial role in the occurrence and development of NPC, and FEZF1-AS1 is the key factor of Wnt/β-catenin signal transmission. Wu et al. [51] proved that after silencing of FEZF1-AS1, the expressions of β-catenin, c-myc and cyclinD1 in GC cells were down-regulated, while the expressions of E-cadherin were up-regulated. It is suggested that FEZF1-AS1 may accelerate tumorigenesis and development of GC by activating the Wnt/β-catenin signal pathway. He et al. [44] found that LncRNA FEZF1-AS1 regulated the Wnt/β-catenin signal pathway in NSCLC.

### PKM2/STAT3 pathway

PKM2 can be used as a multifunctional signaling molecule to promote the proliferation and development of cancer [60]. STAT3 is often overexpressed in tumor cells and tissue samples and regulates the expression of many oncogenes [61]. In CRC, PKM2 is a regulator of STAT3 signaling [62–64] and has been identified as a key downstream target of FEZF1-AS1. Bian et al. [35] found that FEZF1-AS1 knockdown inhibits pyruvate kinase activity, lactic acid production and STAT3 phosphorylation in CRC cells, while PKM2 overexpression saves the above process. Consistent with the above results, Myeloid Cell Leukemia 1 (MCL1), Baculoviral IAP Repeat Containing 5 (BIRC5), Cyclin D1, BCL2 Like 1 (BCL2L1), Cadherin 1 (CdH1), MMP2 and MMP9, as downstream targets of the STAT3 pathway, were also up-regulated in CRC cells with high expression of FEZF1-AS1, and their expression was partially inhibited after PKM2 knockdown. This indicated that FEZF1-AS1 increased the proliferation and metastasis of CRC cells by regulating the PKM2/STAT3 signaling pathway and glycolysis. Again, the 1200–1800 bp region of FEZF1-AS1 can be combined with the A2 domain of PKM2 to improve its stability and facilitate the occurrence and development of CRC.

### FEZF1-AS1 serves as a miRNA sponge

Since the competitive endogenous RNA hypothesis was proposed [65,66], more and more studies have identified the existence of this regulatory effect, despite the controversy. In recent years, there is increasing evidence that lncRNA can play a role as ceRNA in a variety of diseases [67]. FEZF1-AS1 has been detected to be involved in the regulation of the expression of its target genes by binding miRNA in a variety of malignancies. Li et al. [37] reported that FEZF1-AS1 has a binding site of *miR-610*, and FEZF1-AS1 can regulate the expression of AKT3 in MM by binding *miR-610* as ceRNA. Zhou et al. [41] proved that *miR-4443* is a potential binding miRNA of FEZF1-AS1, while there is a potential binding site of *miR-4443* in the 3-utr of NUPR1mRNA. FEZF1-AS1 can promote the expression of NUPR1 by binding *miR-4443*, thus promoting the progression of OS. Zhang et al. [39] verified that in BC, *miR-30a* and FEZF1-AS1 have a total of seven complementary binding sites in the 3′-untranslated region (3′-utr). FEZF1-AS1 binds *miR-30a* as ceRNA, thereby regulating the expression of nanog protein and BC-stem like cells. Qu et al. [21] found that FEZF1-AS1, as ceRNA, can regulate the proliferation and invasion of PC cells by binding to *miR-142* and *miR-133a*. Similarly, Ye et al. [20] accounted that FEZF1-AS1 can combine with *miR-107* to form the FEZF1-AS1/*miR-107*/FEZF1 axis to encourage the progression of pancreatic ductal adenocarcinoma and the Warburg effect.

## Conclusion and future perspectives

FEZF1-AS1 plays an essential role in cell cycle regulation, proliferation, migration, invasion and Warburg effect of various malignancies, and is involved in the regulation of multiple pathways including EMT, WNT and STAT3. Given that almost all cancer features are affected by FEZF1-AS1, it is necessary to mention the other two most important hallmarks of cancer: telomerase activation and inflammation. Telomerase is a ribonuclease that is indispensable to maintain telomere length [68–71], and maintaining sufficient telomere length is requisite for cell proliferation and tumorigenesis [72]. Telomerase reverse transcriptase (TERT) is its core component [73], and the reactivation of TERT and the reconstruction of telomerase activity are necessary for malignant tumor cells to overcome senescence and non-replication. Up to 90% of human cancers have TERT activation, which reactivates TERT through activation of TERT promoter, mutation or other carcinogenic signaling pathways [74–79]. Akincilar et al. [80] quantitative analysis found there is a large number of TERT in human cancer cells and his team [81] revealed long-range chromatin interactions can drive the activation of mutant TERT promoter and promote the development of a variety of tumor cells. Khattar et al. [82] studies have shown that TERT regulated the transcription of pol III (polymerase III) by directly controlling the expression of tRNA, which enhanced the ability of translation and protein synthesis, and thus increased the proliferation ability of many kinds of tumor cells, including BC, liver cancer, glioblastomas, lymphomas and so on. Li et al. [83] detected that activation of mutant TERT promoter by RAS-ERK signaling is a key step in malignant progression of BRAF-mutant human melanomas. With the development of research, more and more scholars have found a large number of cross-acting relationships between lncRNA and telomerase. Liu et al. [84] uncovered LncRNA FOXD2-AS1 functions as a ceRNA to regulate TERT expression by sponging *miR-7-5p* in thyroid cancer. Tan et al. [85] discovered β-catenin-coordinated lncRNA MALAT1 up-regulation of ZEB-1 could enhance the telomerase activity in HGF-mediated differentiation of bone marrow mesenchymal stem cells into hepatocytes. Almost all cancers have an inflammatory response that may be the driving force behind cancer progress. As one of the most typical inflammatory pathways, the nuclear factor-κB (NF-κB) signaling pathway is involved in the development of many human cancers, such as BC and glioblastomas [86–88]. LncRNAs contain modular domains that can directly interact with NF-κB signal proteins to affect the expression or function of oncogenes, tumor suppressor genes, transcription factors and signal transduction pathways [89–91]. Indeed there are many cross-topics between cancer, telomerase activation and inflammation [92–94]. Although there is no direct relationship between FEZF1-AS1 and telomerase activation and inflammation in tumor research, it is not ruled out that FEZF1-AS1 indirectly regulates these hallmarks, the mechanism will be further elucidated with further research. These results suggested that FEZF1-AS1 might become a new tumor marker. However, due to the poor stability of lncRNA *in vitro*, the complex mechanism of regulating gene expression, the low conservatism in different organisms, and the presence of the current research framework mainly in the nucleus, it still needs further efforts to become a meaningful tumor marker for clinical application.

## Abbreviations

AJCC: American Joint Committee on Cancer
AKT3: AKT Serine/Threonine Kinase 3
BC: Breast Cancer
BCL2L1: BCL2 Like 1
BCSC: Breast cancer stem-like cell
CeRNA: competing endogenous RNA
CRC: colorectal cancer
EMT: epithelial–mesenchymal transition
EZH2: Enhancer of Zeste Homolog 2
FEZF1: FEZ Family Zinc Finger 1
FEZF1-AS1: FEZ family Zinc Finger 1-Antisense RNA 1
GC: gastric cancer
HCC: hepatocellular carcinoma
HGF: Hepatocyte Growth Factor
H3K4me2: histone H3lysine-4 di -methylation
JAK: Janus Kinase
LAD: lung adenocarcinoma
LncRNAs: long noncoding RNAs
LSD1: lysine-Specific Demethylase 1
MM: multiple myeloma
MMP2: matrix Metallopeptidase 2
MMP9: matrix Metallopeptidase 9
NcRNAs: noncoding RNAs
NF-κB: nuclear Factor-κB
NPC: nasopharyngeal carcinoma
NSCLC: non-small-cell lung cancer
OS: osteosarcoma
PC: pancreatic cancer
PKM2: pyruvate Kinase M 2
qRT-PCR: quantitative real-time PCR
STAT3: signal transducer and activator of transcription 3
TERT: telomerase reverse transcriptase
TNM: Tumor Lymph Node Metastasis
VEGF: Vascular Endothelial Growth Factor
ZO-1: zonula occluden-1
